# miRNA‐122‐5p in POI ovarian‐derived exosomes promotes granulosa cell apoptosis by regulating BCL9

**DOI:** 10.1002/cam4.4615

**Published:** 2022-03-01

**Authors:** Xiujuan Zhang, Ruihong Zhang, Jing Hao, Xiaoyan Huang, Ming Liu, Mengxiao Lv, Chan Su, Yu‐Lan Mu

**Affiliations:** ^1^ Department of Gynecology and Obstetrics Shandong Provincial Hospital Affiliated to Shandong First Medical University Jinan China; ^2^ Key Laboratory of the Ministry of Education for Experimental Teratology, Department of Histology and Embryology School of Medicine, Shandong University Jinan China; ^3^ Shandong Maternal and Child Health Care Hospital Jinan China; ^4^ Shandong University of Traditional Chinese Medicine Jinan China

**Keywords:** BCL9, exosome, miR‐122‐5p, ovarian granulosa cell, premature ovarian insufficiency

## Abstract

This study is to explore the therapeutic effect and potential mechanisms of exosomal microRNAs (miRNAs) derived from the ovaries with primary ovarian insufficiency (POI). The POI mouse model was established by intraperitoneal injection of cyclophosphamide (CTX) and busulfan. The apoptosis of granulosa cells (GCs) incubated with exosomes extracted from ovarian tissues of control and POI groups was analyzed by flow cytometry. Then, high‐throughput sequencing was performed to detect the difference of miRNAs profile in ovarian tissue‐derived exosomes between the control and POI mice. The effect of differential miRNA on the apoptosis of CTX‐induced ovarian GCs was analyzed by flow cytometry. The results showed that POI mouse model was successfully established. Exosomes extracted from ovarian of normal and POI group have different effects on apoptosis of GCs induced by CTX. miRNA‐seq found that exosomal miR‐122‐5p in POI group increased significantly. miR‐122‐5p as the dominant miRNA targeting BCL9 was significantly upregulated in ovarian tissues of chemotherapy‐induced POI group. Exosomes derived from the ovaries in the control group and miR‐122‐5p inhibitor group attenuated the apoptosis of primary cultured ovarian GCs. In conclusion, exosomal miR‐122‐5p promoted the apoptosis of ovarian GCs by targeting BCL9, suggested that miR‐122‐5p may function as a potential target to restore ovarian function.

## Graphical abstract

This study is to explore the therapeutic effect and potential mechanisms of exosomal microRNAs (miRNAs) derived from ovarian on primary ovarian insufficiency (POI). The POI mouse model was established by intraperitoneal injection of cyclophosphamide (CTX) and busulfan. The apoptosis of granulosa cells (GCs) incubated with exosomes extracted from ovarian tissues of control and POI groups were analyzed by flow cytometry. Then, high‐throughput sequencing was performed to detect the difference of miRNA profile in ovarian tissue‐derived exosomes between the control and POI mice. Further analyzed the effect of miR‐122‐5p on the apoptosis of CTX‐induced ovarian GCs by flow cytometry. The POI mouse model was successfully established. Exosomes extracted from ovarian of normal and POI group have different effects on apoptosis of GCs induced by CTX. miRNA‐seq found that miR‐122‐5p in POI group increased significantly. miR‐122‐5p as the dominant miRNA targeting BCL9 was significantly upregulated in ovarian tissues of chemotherapy‐induced POI group. Exosome derived from the ovarian in the normal group and miR‐122‐5p inhibitor inhibited the apoptosis of primary cultured ovarian GCs. In conclusion, exosomal miR‐122‐5p promoted the apoptosis of ovarian GCs by targeting BCL9, suggested that miR‐122‐5p may function as a potential target to restore ovarian function.

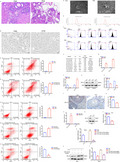

## 1 INTRODUCTION

Primary ovarian insufficiency (POI) or premature ovarian failure (POF) is a heterogeneous gynecological endocrine disease caused by follicular failure or dysfunction. It is characterized by elevated serum gonadotropin level and absence of residual follicles in the gonads, which are associated with oligomenorrhea, amenorrhea, subfertility, and even infertility. The clinical manifestations are hot flashes, sweating, and decreased libido.^1^ In POI, the process of follicle depletion occurs at a young age (generally taken as 40 years old).^2^ The incidence of POI in the general population is estimated to be 1%.^3^ In addition, the incidence of POI in women is increasing with age. POI is divided into two types: primary and secondary POI. Primary POI is generally caused by heredity,^4^ immune, inflammation, enzyme metabolism disorder, and so on. While unfortunately, the primary cause of POI remains unknown^5^; Secondary POI is generally caused by surgery, radiotherapy, chemotherapy, and other exposure to toxic substances.^6^ High‐dose radiotherapy and chemotherapy can alleviate cancer in more than 90% of young females, while they have adverse effects on ovarian function and reproductive capacity.^7^ The main mechanisms of chemotherapy leading to ovarian dysfunction can be divided into two categories: A. Dysfunction due to direct toxicity to oocytes and ovarian GCs. B. Damage to growing oocytes indirectly leads to over‐activation and depletion of primitive oocytes.^8^ Ovarian GCs are a layer of parietal cells around the surface of follicles, which support the formation and development of follicles and have the ability to secrete glandular hormones to maintain ovarian function. Aberrant ovarian GC death leads to follicular atresia and decreases the number of follicles.^9^ Studies have shown that cyclophosphamide (CTX) does not directly act on oocytes, but induces senescence and apoptosis of GCs in growing follicles, which leads to secondary loss of oocytes.^10^ Therefore, inhibiting apoptosis of GCs is the key to prevent chemotherapy‐induced POI.

Studies have found that the application of stem cells can rejuvenize follicle and GCs regeneration. These effects may be that mesenchymal stem cells directly migrate to the damaged ovary and differentiate into follicles in the ovarian microenvironment, to promote the recovery of reproductive endocrine and inhibit the apoptosis of GCs, suggesting that transplantation of mesenchymal stem cells can repair the ovarian structure and improve ovarian function.^11^ However, stem cell‐based therapy still faces some challenges, including transplant rejection, tumor transformation, limited sources, ethical issues, etc., which lead to its limited clinical application.^12^ Autologous healthy ovarian tissue transplantation can improve the endocrine level and improve ovarian function, and can effectively restore the reproductive ability of ovarian mice damaged by chemotherapy.^13^ Based on this, researchers who were focused on the effective substances in cells found that exosomes derived from cells can be used to treat some diseases. Compared with cells, exosomes have the advantages of low immunogenicity, non‐tumorigenicity, high clinical safety, and low ethical risk.^14^ Exosomes play a key role in embryonic development, gametogenesis, and fertilization. Many studies have found that exosomes derived from cells can significantly restore the estrous cycle of POI mice, increase the number of basal and antral follicles, inhibit the apoptosis of ovarian GCs. However, its mechanism remains unclear.

Exosomes belong to extracellular vesicles, which are uniform membranous vesicles with a diameter of 30–150 nm. The outer membrane of the vesicles is fused with the cell membrane and released out of the cell. Exosomes carry signal molecules such as mRNA, microRNA (miRNA), circRNA, lncRNA, and protein, and complete the signal transmission between adjacent or distant cells utilizing target cell internalization, ligand‐receptor interaction or lipid membrane fusion, and then play important physiological or pathological functions, including coagulation, inflammation, cell expansion, neuronal communication, and tumorigenesis, etc.^15^, ^16^ Furthermore, both normal cells and tumor cells can secrete exosomes.^17^ Chemotherapy can alleviate malignant tumors, and also promote their secretion of different exosomes, and their compositions are significantly diverse. For example, annexin 6 and miRNA molecules play an important role in tumor cell metastasis, tumor drug resistance, and other non‐tumor cell lesions.^18^, ^19^ It is found that the changes of exosomes and their substances in pathogenic tissues provide a theoretical basis for exploring new diagnostic features, mechanisms, and treatment measures. Therefore, it is a new idea to study the changes of exosomal components in normal or chemotherapy mice ovarian tissue for the treatment of POI.

As an important part of exosomes, miRNA plays an important regulatory role in cell growth, differentiation, communication, and migration, as well as maintaining homeostasis and pathological changes by binding to specific targets.^20^, ^21^ Previous studies have reported that miRNA is differentially expressed in plasma and tissue of POI patients, compared to normal people, which plays an important role in the pathogenesis, diagnosis, and efficacy evaluation of POI. In this study, we cultured ovarian tissues and ovarian GCs of female mice, isolated exosomes from ovarian tissues, identified differentially expressed miRNAs in the ovarian exosomes of CTX‐treated mice. Then, we proved the protective effect of normal ovarian exosomes on follicles and revealed that miR‐122‐5p inhibitor protected ovarian GCs apoptosis induced by CTX through target gene BCL9, which provided the cytological basis for further in vivo experiments.

## 2 Materials and methods

### 2.1 Animals

Female ICR mice (Charles River, Beijing, China) have high survival rate and strong reproductive ability and were used as experimental animals, which were raised at room temperature, humidity 45%–55% and illumination time 12 h. The study was conducted according to the guidelines of the Declaration of Helsinki. All experimental research steps involving these animals were subject to approval by the Medical Ethics Committee of the Shandong Provincial Hospital Affiliated to Shandong First Medical University (approval no.2019132, 14 November 2020).

### 2.2 Establishment of mouse POI model

The female ICR mice aged from 6 to 8 weeks, were injected intraperitoneally (I.P.) with CTX (20 mg per kg of body weight daily) and busulfan (8.75 mg per kg of body weight daily) (Otsuka America) for 2 weeks. In the control group, ICR female mice were injected with phosphate‐buffered saline (PBS) at the same dose intraperitoneally. Two weeks later, the animals were sacrificed, tissues were collected.

### 2.3 Ovarian histological characteristics

Immediately after mice were sacrificed, the bilateral ovaries were dissected and fixed in 75% alcohol for 5 min. In the ultra‐clean table, cut the abdominal wall, take out the bilateral ovaries, quickly put them into a 35‐mm Petri dish containing PBS (preheated at 37°C), fix them with 4% paraformaldehyde (Solarbio). After 24 h of fixation, increasing concentrations of ethanol dehydrated the ovarian tissue, xylene (Solarbio, Beijing, China) clarified the ovarian tissue, and the tissues were paraffin‐embedded. The paraffin was sectioned (4 μm thick) for hematoxylin and eosin staining to observe the morphological structure of the mouse ovaries under an optical microscope.

### 2.4 Isolation, culture, and different treatment of ovarian GCs

Three‐week‐old ICR female mice were intraperitoneally injected with 10 IU of pregnant mare serum gonadotropin (Jinke). After 24 h, these mice were euthanized, and their ovaries were isolated mechanically. Ovarian GCs were obtained by puncturing follicles under a stereomicroscope. Then complete medium (DME/F‐12 1:1 [1×], 10% FBS, 3.7 mg/ml NaHCO_3_, and 1% Penicillin–Streptomycin) was added to ovarian GCs and incubated in a 37°C in a humidified atmosphere with 5% CO_2_. GCs were inoculated into six‐well plates with 2 × 10^5^ cells per well. GCs with an adhesion rate of 50%–60% were selected for follow‐up experiments and divided into two groups: one group was added with 2 μM CTX^22^; the other group added the same amount of PBS as the control group; cultured for 24 h to induce apoptosis of GCs.

### 2.5 Isolation and identification of ovarian tissue‐derived exosomes

ICR female mice aged 6 weeks were selected. After 2 weeks of treatment with the above chemotherapy regimen, the mice were sacrificed, ovarian tissues were collected, and cultured in DMEM containing 100 U/ml Penicillin and 100 μg/ml Streptomycin in a humidified incubator of 5% CO_2_ at 37 °C. After 24 h, exosomes were extracted by gradient ultracentrifugation. The collected supernatant of ovarian tissue was centrifuged at increasing speeds of 300× g for 10 min, 2000× g for 10 min, 3000× g for 15 min to remove cell debris with 0.22 μm filter, and 10,000× g at 4°C for 30 min to remove sediment and debris. Then, the supernatant was centrifuged twice at 120,000× g for 80 min in clear ultracentrifuge tubes, resuspend the precipitate with 200 μl of particle‐free PBS. To confirm the successful isolation of the separated and purified exosomes, western blot was used to identify the characteristic marker proteins of the exosome, including CD63 and CD81 (Affinity). Then observing the difference in quantity, shape, and size of exosomes from two groups under transmission electron microscopy (TEM), and taking pictures.

### 2.6 Effect of exosomes on apoptosis of ovarian GCs

Exosomes extracted from ovarian tissues were co‐cultured with ovarian GCs. The GCs in the six‐well plate were divided into two groups: one group was added PBS as the control group; the other group was added with 2 μM CTX. Cultured for 24 h to induce apoptosis of GCs. CTX group was then divided into two groups, were added exosomes derived from ovarian tissue of POI mice and normal mice, respectively. Cells were gathered after 48 h.

### 2.7 Flow cytometry assay

According to the manufacturer's procedure, CTX‐induced granulosa cell (GC) apoptosis was determined by FACS using FITC Annexin V/PI Apoptosis Detection Kit. In short, cells were seeded in six‐well plates at a density of 5 × 10.^5^ After 2 days, the cells were washed three times with cold PBS and stained with FITC Annexin V and propidium iodide (PI) for 15 min at room temperature using the Annexin V‐FITC Apoptosis Detection Kit. Then we analyzed the stained cells by fluorescence‐activated cell sorting (FACS).

### 2.8 Exosome‐derived miRNA high‐throughput sequencing (miRNA‐seq)

Exosomes were extracted from ovarian tissues of normal group and POI group, centrifuged at 2000 *g* for 5 min, and then resuspend the precipitate with 200 μl of particle‐free PBS. RNA was extracted separately and sent to the company (RiboBio) for miRNA high‐throughput sequencing.

### 2.9 Quantitative real‐time polymerase chain reaction

Total RNA was extracted using Trizol reagent (Invitrogen), cDNA was synthesized by reverse transcription (Thermo Fisher Scientific). SYRB Green PCR Kit (CWBIO) were used for quantitative real‐time polymerase chain reaction (qRT‐PCR) analysis. All qRT‐PCR reactions were tested in triplicates with the following thermocycling conditions: 95°C for 10 min, followed by 40 cycles at 95°C for 15 s and 60°C for 30 s and 72°C for 32 s on a CFX96™ Real Time PCR Detection system (Bio‐Rad). The relative levels of gene expression were quantified using 2^−ΔΔCt^ method. GAPDH was used as an internal control for all samples. The BCL9 primer sequence as follows: 5′‐CGGGGACTTGGATGTGAG‐3′ and 5′‐TTTCAAGAGGTCCACTCCGC‐3′.

### 2.10 Western blot

Ovarian tissue‐derived exosomes were lysed in RIPA buffer with protease inhibitor cocktail (Sigma). The lysates were resolved on SDS–PAGE and transfered onto 0.22 μm PVDF membrane (Millipore), and then blocked with 5% skim milk for 2 h at room and then incubated with primary antibodies at 4°C overnight. Primary antibodies at the following dilutions: BCL9 (1:1000, ab37305; Abcam), β‐actin (1:1000, #4970; CST). Subsequently, incubated with horseradish peroxidase‐conjugated secondary antibody (1:5000 dilution) for 2 h at room temperature. A complex of primary and secondary antibodies labeled was detected by an enhanced chemiluminescence (Millipore) system followed by exposure to Amersham Imager 600.

### 2.11 Immunohistochemistry

Ovarian tissue of mice preserved in 4% paraformaldehyde was dehydrated and embedded in paraffin. The ovarian tissue was cut into 4 μm thick slices, dewaxed in gradient ethanol and xylene, and then immersed in the PBS. After rehydration, the membrane‐breaking solution broke the cell membrane, then degreased with 3% hydrogen peroxide methanol. Then the membrane was incubated with primary antibody for 12 h at 4°C in a humid environment. Subsequently, the membrane was incubated with horseradish peroxidase‐conjugated secondary antibody for 50 min at room temperature. Ovarian slices were stained with a DAB kit (Solarbio), counterstained with hematoxylin, and sealed with neutral resin after dehydration. The difference between the control group and the administration group was observed under an optical microscope. At last, immunoblot densitometry was performed using Image J software.

### 2.12 Effect of miR‐122‐5p on apoptosis of GCs

To further clarify whether miR‐122‐5p can protect ovarian GCs from CTX injury, 2 μM CTX was added to GCs to induce apoptosis of cells; 24 h after culture, the group was set up into four groups, followed by adding miR‐122‐5p mimic NC, miR‐122‐5p mimic, miR‐122‐5p inhibitor NC, and miR‐122‐5p inhibitor (RiboBio). Twenty‐four hours later, the cells were collected, and the effects of miR‐122‐5p mimic and inhibitor on apoptosis of GCs was detected by PI/Annexin‐V flow cytometry. The transfection efficiency was assessed by qRT‐PCR and western blot at 24 h after transfection.

### 2.13 Statistical analysis

GraphPad Prism 8 (GraphPad Software, lnc.), Image J (Image J software, lnc.), and SPSS 22.0 (SPSS software, lnc.) were used for statistical analysis. Comparisons between groups were analyzed by the *t*‐test and *X* ^2^ test. Data are presented as the mean ± SD of three independent experiments. Results were considered statistically significant for *p* < 0.05.

## 3 Results

### 3.1 Effect of chemotherapy drugs on mouse ovary and GCs

**FIGURE 1:**
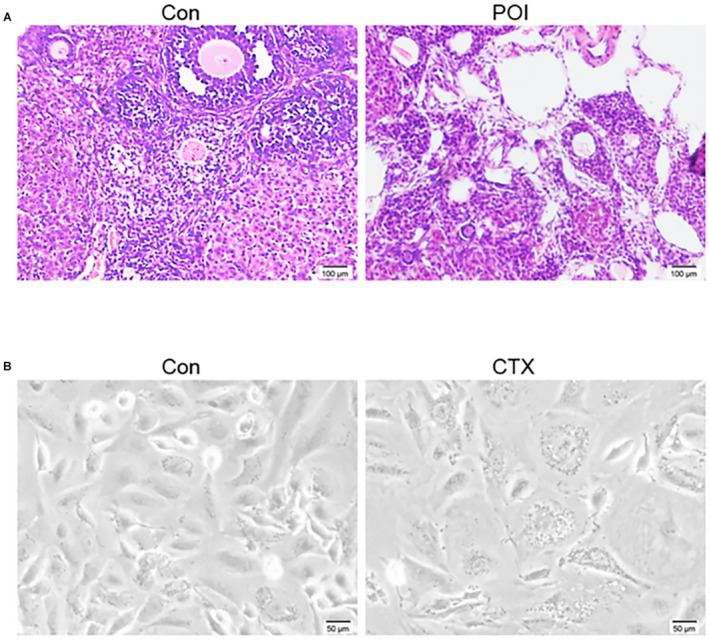
Typical characteristics of the ovary and granulosa cells (GCs) of normal and mice treated with chemotherapy drugs. (A) Histological analysis of ovarian sections of 6 weeks old mice after 2 weeks of treatment with (right) or without (left) cyclophosphamide (CTX) and busulfan, a representative hematoxylin and eosin staining of 15 mouse ovaries. Scale bar: 100 μm; (B) A representative GCs morphology of normal (left) or with CTX treatment (right), the cells were polygonal and fiber‐like; compared with the control group, the number of ovarian GCs in the CTX group was significantly reduced. Scale bar: 50 μm

After 2 weeks of treatment with CTX and busulfan, the POI model of ICR female mice was successfully established. The ovarian morphological characteristics between the control group and the POI group were compared. In the control group, the mouse ovaries had large and abundant follicles, abundant follicular fluid, and multiple corpus luteum. While in the POI group the ovaries had few and mostly primitive or initial follicles, and the atresia follicles and interstitium were increased due to GCs injury (Figure 1A). In the cell cultural model, microscopic observation showed that 48 h after inoculation with CTX at the concentration of 2 μM, the treated GCs adhered and grew in the visual field, showing polygonal, fibrous structures, and pseudopodium. Compared with the control group, CTX treatment also reduced the number of the cells (Figure 1B).

### 3.2 Extraction and characterization of ovarian exosomes

**FIGURE 2:**
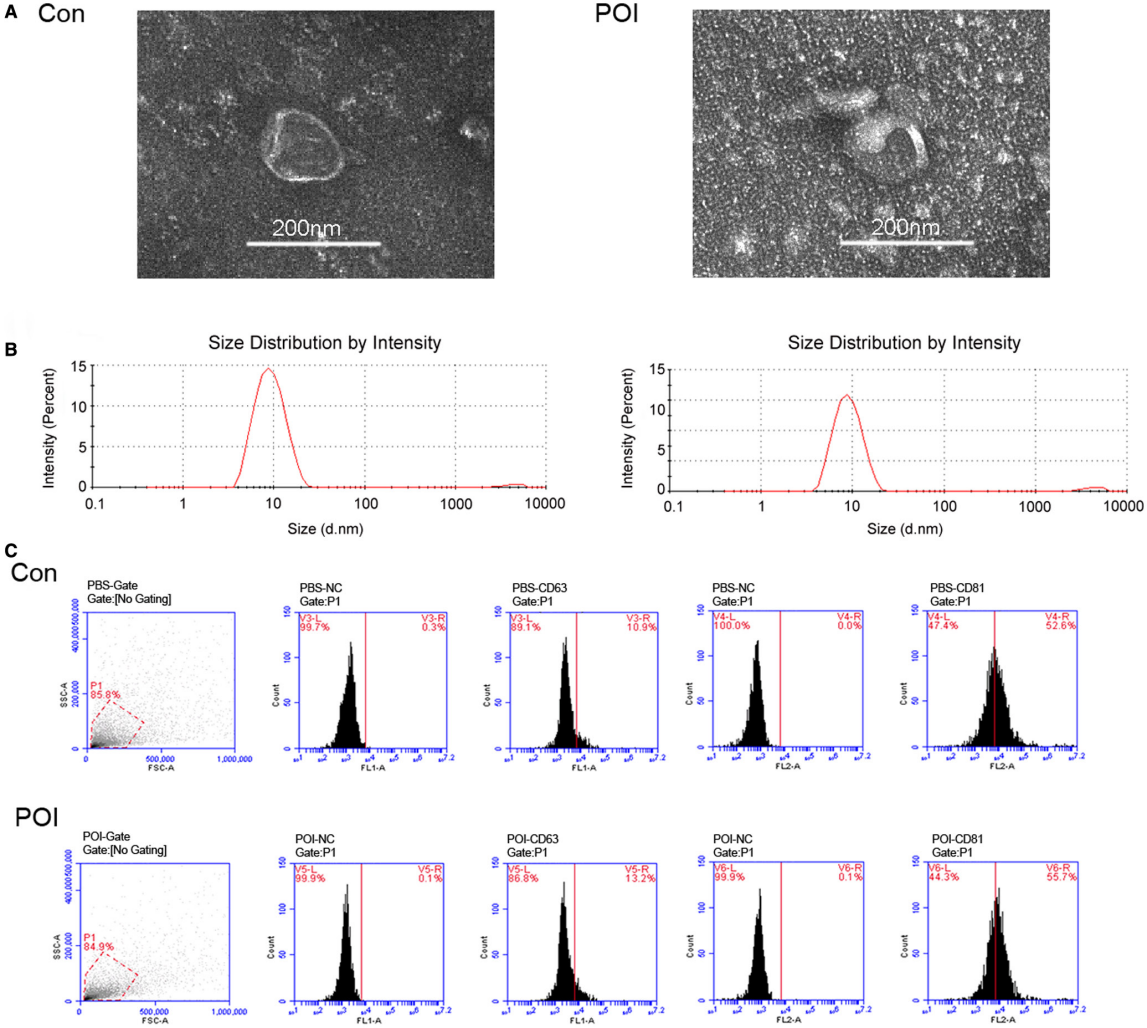
Isolation and characterization of ovarian exosomes. (A) Electron microscopy image of exosomes isolated from ovarian tissues of the control group and POI groups. Scale bar: 200 nm. (B) Detection of exosome particle size in each group. (C) FACS analysis of exosome surface marker protein. They were positive for CD63 and CD81

Exosomes were extracted from ovarian tissue using gradient ultracentrifugation. Ovarian tissue‐derived exosomes were characterized by morphological observation and marker protein detection. By TEM, membranous vesicles were uniform size, round or oval shape, with clear margins, and double lipid membranes surrounding them (Figure 2A). The morphological analysis showed that the size distribution of exosome intensity was 50–150 nm (Figure 2B). There was no significant difference in the shape and size of ovarian exosomes between the control and the POI groups. FACS analysis showed positivity of the typical exosomal surface marker proteins CD63 and CD81 (Figure 2C).

### 3.3 Effects of ovarian exosomes on CTX‐damaged ovarian GCs

**FIGURE 3:**
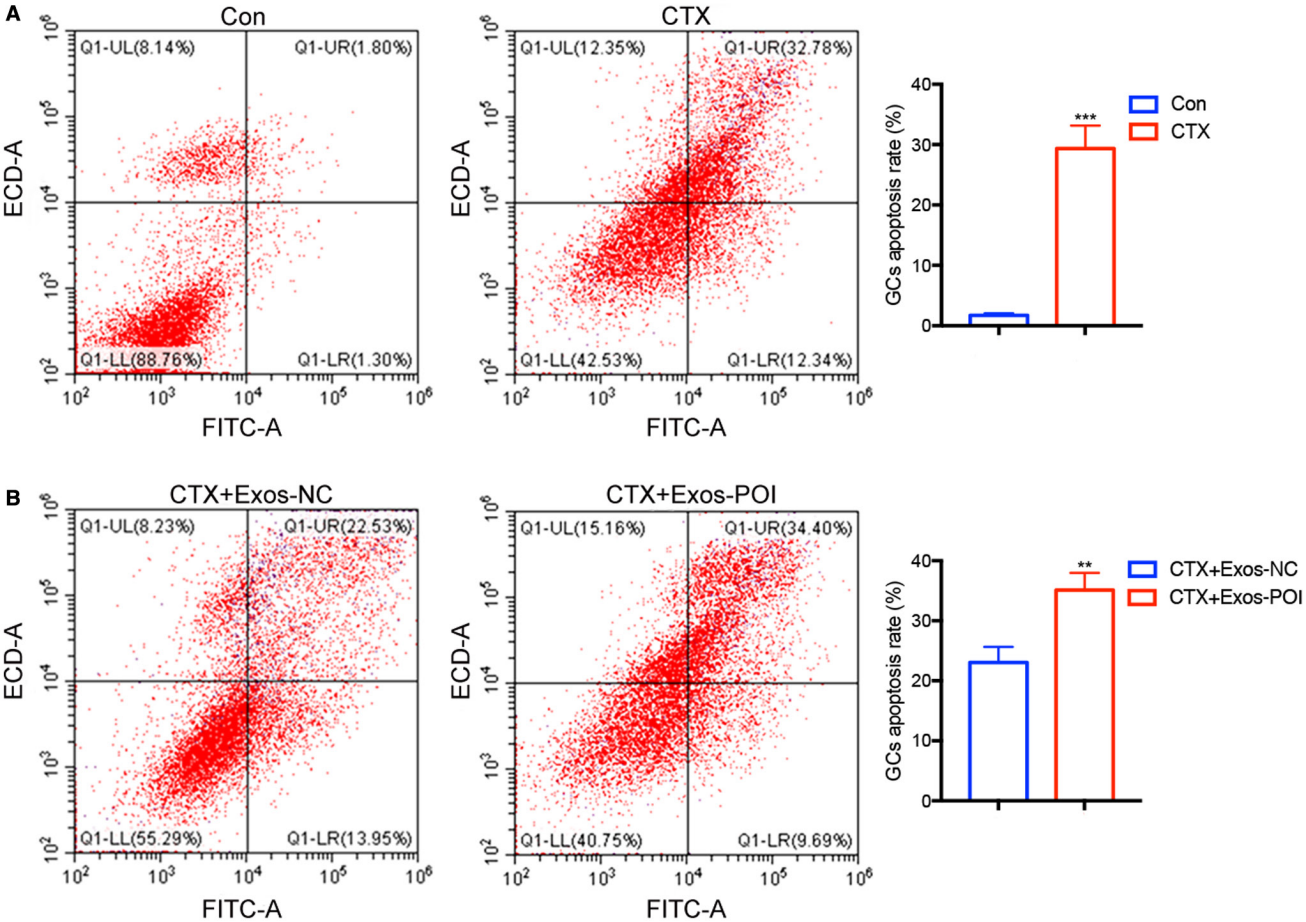
The effect of ovarian exosomes extracted from normal and POI mice on apoptosis of cultured GCs. (A, B) Exosomes protect against CTX‐induced injury of GCs and promote resistance to cell apoptosis in vitro based on FACS. Groups were cultured for 48 h, through PI/Annexin‐V double staining and FACS analysis. CTX+ Exos‐NC, CTX+ exosomes from normal mice ovarian tissue. CTX+ Exos‐POI, CTX+ exosomes from POI mice ovarian tissue

To further assess the therapeutic role of exosomes derived from normal ovarian tissue in CTX‐damaged ovarian GCs, CTX‐injured cells were co‐cultured with ovarian exosomes extracted from the control group and the POI group, respectively. Flow cytometry analysis showed that CTX promoted apoptosis of GCs (*p* < 0.001, Figure 3A). The proportion of apoptotic cells in the exosome‐POI group was higher than that in the exosome‐NC group, indicated that ovarian exosomes extracted from POI mice promoted apoptosis of GCs, while the control ovarian tissue‐derived exosomes ameliorated the status of GCs damaged by CTX (*p* < 0.01, Figure 3B). The data indicate that that healthy ovarian tissue‐derived exosomes could reverse the course of POI in the CTX mouse model.

### 3.4 Identification of miRNAs in exosomes of normal mice and POI models

**FIGURE 4:**
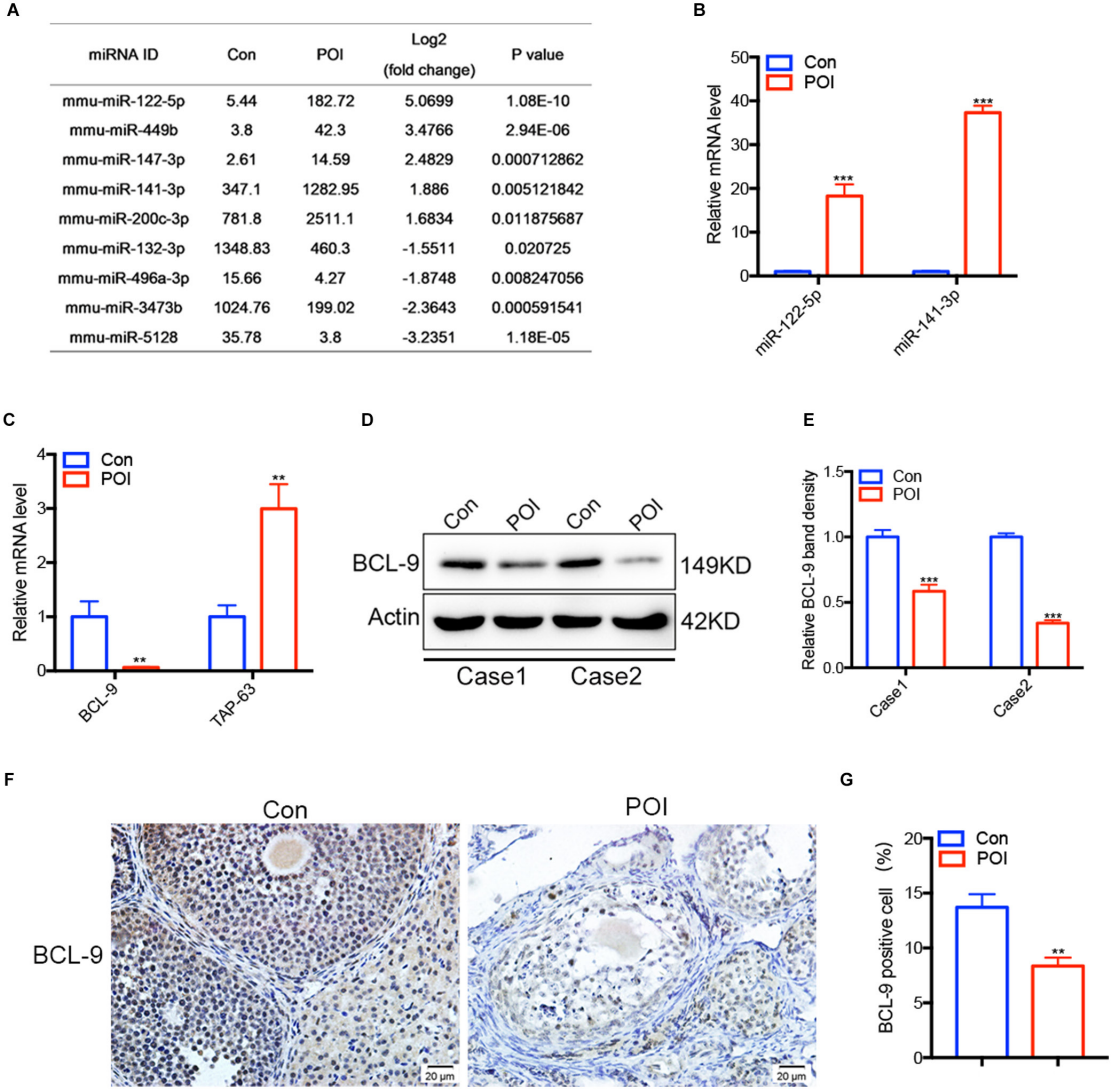
Differentially expressed miRNAs in ovarian exosomes and their target genes in ovarian tissues. (A) The levels of miRNAs in ovarian exosomes in the control group and the POI group. (B) The miR‐122‐5p and miR‐141‐3p expression in each group were detected by quantitative real‐time polymerase chain reaction (qRT‐PCR). (C) The BCL9 and TAP‐63 expression in each group were determined by qRT‐PCR. BCL9 in ovarian tissue between the two groups was determined by western blot (D) and gray scale analysis (E). Immunohistochemistry (F) and Image J analyses (G) were used to verify the difference of BCL9 in ovarian tissue between the two groups. *p < 0.05, **p < 0.01, ***p < 0.001

In the research, it was found by high‐throughput sequencing that the expression profiles of miRNAs in ovarian exosomes of the control group and the POI group were markedly different. There were 30 miRNAs upregulated or downregulated by more than two times, among which miR‐122‐5p related to apoptosis is upregulated by more than 32‐times in the ovarian exosomes of the chemotherapy group (Figure 4A). To further determine the role of miRNAs in CTX‐induced POI, we first assessed the transcription level of miR‐122‐5p and miR‐141‐3p by using qRT‐PCR, the result showed that their expression levels in ovarian exosomes of the POI group were significantly higher than those in the control group (Figure 4B). We further explored their downstream targets in signal pathways were also altered, the results of qRT‐PCR and western blot assay showed that BCL9, the target molecule of miR‐122‐5p, was indeed significantly downregulated in the ovary of the POI group compared with the control group, while TAP‐63 is the opposite. (Figure 4C–E). Immunohistochemistry analysis further confirmed that the expression of BCL9 was downregulated in the ovarian tissues of the POI mice (Figure 4F,G).

### 3.5 miR‐122‐5p regulates apoptosis of ovarian GCs

**FIGURE 5:**
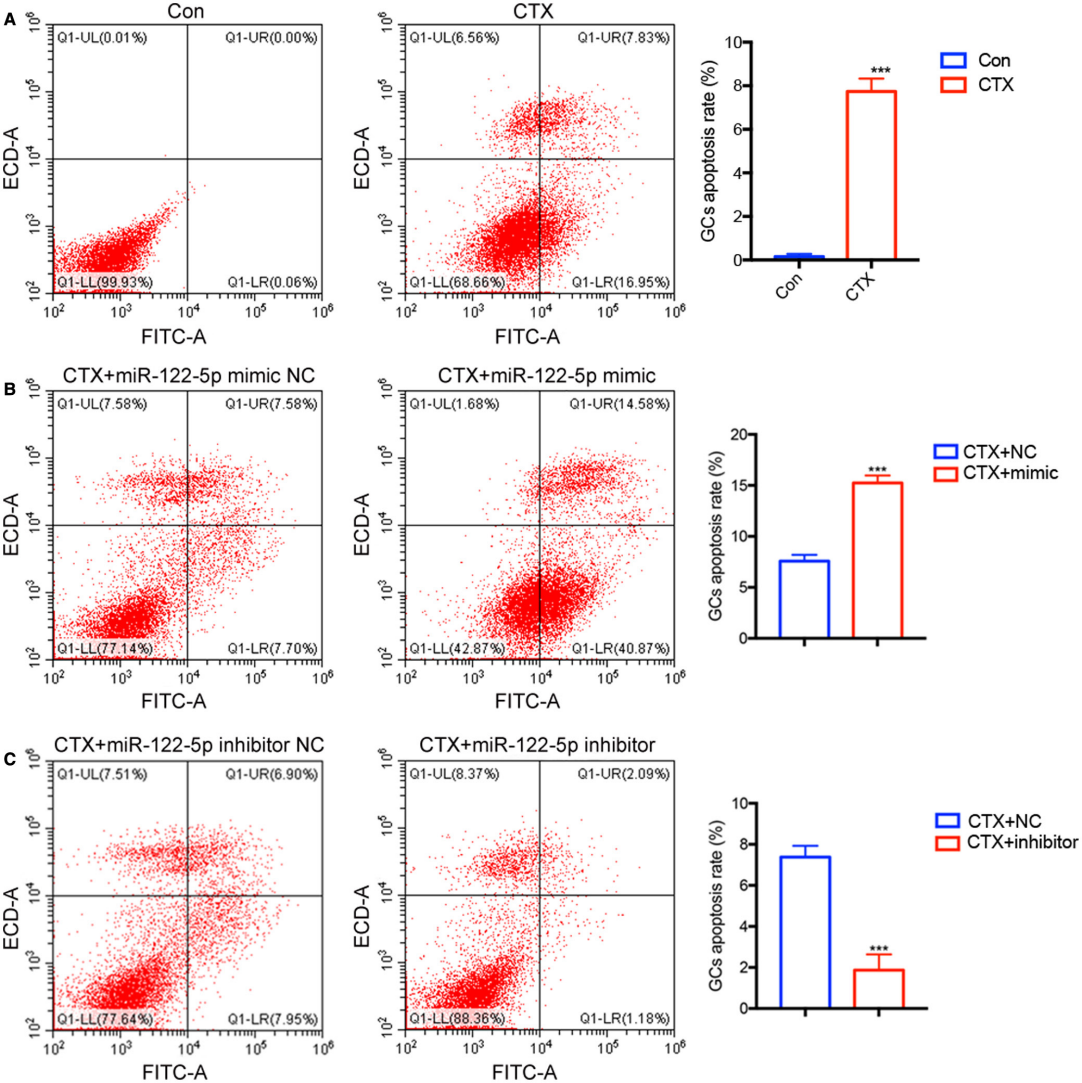
The effect of miR‐122‐5p on apoptosis of ovarian GCs. Flow cytometry analysis was used to analyze GCs apoptosis after increasing and reducing the expression of miR‐122‐5p in the group. (A) CTX‐induced apoptosis of ovarian GCs. (B) miR‐122‐5p mimic promoted apoptosis of GCs. (C) miR‐122‐5p inhibitor weakened the apoptosis of GCs

To further study the effect of exosomal miR‐122‐5p on ovarian GCs, miR‐122‐5p mimics, and inhibitors were selected for further analysis. miR‐122‐5p mimic NC, miR‐122‐5p mimic, miR‐122‐5p inhibitor NC, and miR‐122‐5p inhibitor were co‐cultured with CTX‐damaged ovarian GCs. Flow cytometry analysis showed that CTX promoted apoptosis of GCs (Figure 5A). The proportion of apoptotic cells in the miR‐122‐5p mimic group was higher than that in the miR‐122‐5p mimic NC group, indicated that miR‐122‐5p promoted apoptosis of GCs (Figure 5B). Compared with the control group, the proportion of apoptotic cells in the miR‐122‐5p inhibitor NC group increased, while the miR‐122‐5p inhibitor reduced the apoptosis of GCs (Figure 5C). The results indicate that miR‐122‐5p inhibitor inhibits apoptosis of ovarian GCs injured by CTX.

### 3.6 miR‐122‐5p inhibitor inhibits apoptosis of GCs via BCL9

**FIGURE 6:**
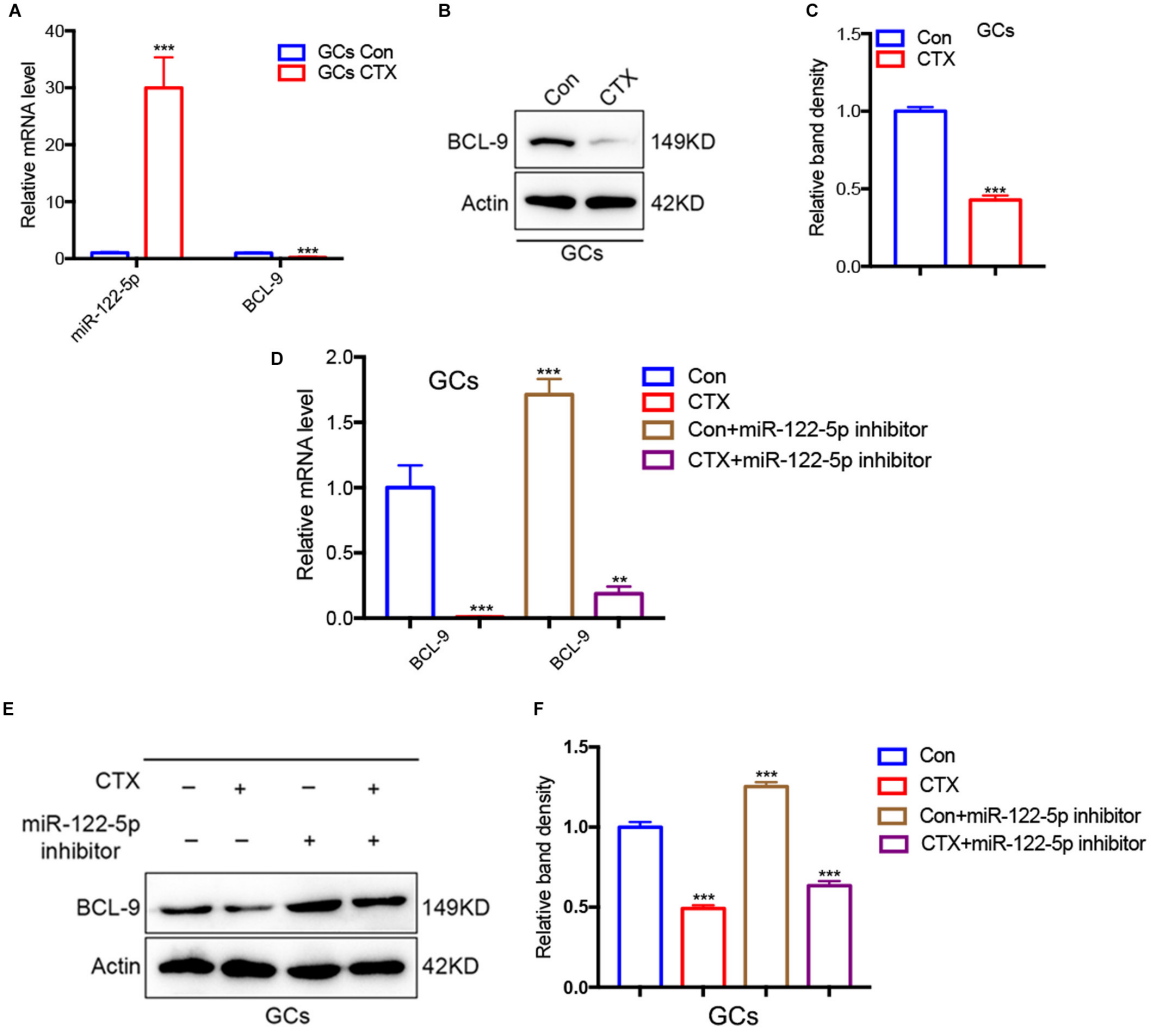
miR‐122‐5p inhibits apoptosis of GCs via BCL9. (A) The levels mRNA of miR‐122‐5p and BCL‐9 in GCs treated with or without CTX were assessed by quantitative real‐time polymerase chain reaction (qRT‐PCR). (B) Western blots of BCL‐9 in GCs treated with or without CTX. (C) Quantification of the western blots in (B) of BCL‐9 in GCs treated with or without CTX. (D) qRT‐PCR analysis of the levels of BCL‐9 in GCs incubated with miR‐122‐5p inhibitors. (E, F) Western blot was performed to detect the expression of BCL9 in GCs after adding the miR‐122‐5p inhibitor. Data are expressed as mean ± SD

To further evaluate the mechanism by which the ovarian exosomal miR‐122‐5p promotes apoptosis of ovarian GCs, the transcription levels of BCL9 and miR‐122‐5p in ovarian GCs, the level of miR‐122‐5p in ovarian GCs of the CTX group was significantly upregulated compared with that in the control group, while the expression of BCL9 was the opposite (Figure 6A). Western blot analysis showed that CTX significantly inhibited the expression of BCL9 in ovarian GCs (Figure 6B,C). In order to further check if miR‐122‐5p inhibitor could reverse the detrimental effect caused by CTX treatment, CTX‐damaged GCs were incubated for 2 days in cell culture medium containing the miR‐122‐5p inhibitor and analyzed by qRT‐PCR (Figure 6D) and western blot assay indicated that BCL‐9 was upregulated (Figure 6E,F). The data confirmed that (1) miR‐122‐5p is upregulated, (2) the role of the signal pathway of miR‐122‐5p/BCL9 in the course of POI and its therapeutic application in chemotherapy‐induced POI, and (3) mir‐122‐5p inhibitor can protect GCs from apoptosis by upregulating BCL9 expression.

## 4 DISCUSSION

Currently, in a survey of cancer patients in 25 countries, the 5‐year net survival rate of women diagnosed with cancer between 2010 and 2014 is 85% or higher, and the survival rate of young cancer patients is significantly higher than that of elderly patients.^23^ In China, the survival rate of breast cancer patients under 35 years old is 90.91%, and that of patients aged 35–45 years old is 57.84%.^24^ For POI induced by chemotherapy, the damage to the ovary is different according to the scope of chemotherapy and radiotherapy, the age of the patient, the type of medication, and the dosage. Among the chemotherapy drugs, the alkylated drugs do the greatest harm to the ovary. Therefore, in this experiment, we selected common clinical animal models of POI induced by CTX combined with busulfan as well as a cell model of ovarian GCs injury induced by CTX in vitro. Studies have shown that intraperitoneal injection of CTX combined with busulfan can cause endocrine dysfunction and ovarian tissue damage in mice, leading to ovarian failure.^25^

Chemotherapy may cause irreversible ovarian damage, so the fertility preservation of women of childbearing age and adolescent women during the anticancer period is extremely significant.^6^ However, fertility preservation methods recommended by the American Reproductive Medicine Association are to preserve the couples' embryos and the female oocytes.^26^ Although hormone replacement therapy can be used for a long time to relieve symptoms, it can not prevent its occurrence and progress.^27^ Besides, ovarian tissue transplantation and stem cell transplantation can not cure POI, due to their poor prognosis and uncontrollable long‐term results.^28^ Therefore, it is urgent to find a new clinical treatment strategy for POI patients. To date, increasing evidence shows that the therapeutic activities of stem cells and ovarian cells through the paracrine process, which leads to a novel concept of “cell‐free” therapy.

Based on reports, exosomes are a new way of interaction between cells. Exosomes are nano‐scale biological lipid double‐membrane vesicle carriers with stable biological characteristics, and their molecular markers are integrins, CD24, CD63, CD81, CD91, HSPA8, and HSC70. The vesicles contain MHC I and MHC II, lipids, adhesion proteins, and nucleic acid components, which release vesicles (exosomes) and combine with cell membranes in a calcium‐dependent manner.^15^ In recent years, studies have confirmed that exosomes derived from cells are effective in treating diseases such as liver failure, heart failure, and ovarian cancer by transferring proteins and RNA. In addition, exosomes also play a key role in various aspects of dysplasia, including embryonic development, gametogenesis, and fertilization. Exosomes derived from human umbilical cord mesenchymal stem cells^29^ and bone marrow mesenchymal stem cells^30^ can significantly upregulate the level of BCL‐2 and downregulate the level of Caspase‐3 in the cisplatin‐induced POI model, suggesting that exosomes can protect ovarian GCs from apoptosis induced by chemotherapy drugs. In our study, we investigated the role of ovarian tissue‐derived exosomes in CTX‐induced apoptosis of GCs in mice. The results showed that the exosome derived from ovarian tissue in normal mice significantly weakened the apoptosis of ovarian GCs, while the exosome derived from ovarian tissue in POI mice deteriorated the apoptosis of ovarian GCs. The results indicate that the exosomes derived from normal ovarian tissues have a protective effect on GCs injury.

The abnormal expression of miRNAs is closely related to many diseases. For example, the exosomal miR‐205 from cancer cells can regulate angiogenesis and tumor metastasis^31^; miR‐106a is closely related to ovarian development; Transfection of anti‐miR‐106a or pre‐miR‐591 enhances apoptosis of ovarian cancer cell SKpac, and inhibits cell migration and proliferation.^32^ Researchers have found that the exosome miR‐10a derived from amniotic fluid stem cells,^33^ the exosome miR‐644‐5p derived from bone marrow mesenchymal stem cells^30^ and miR‐144‐5p^22^ can inhibit the apoptosis of damaged GCs, prevent follicular atresia after chemotherapy, and improve ovarian failure caused by chemotherapy. In addition, human umbilical cord mesenchymal stem cells miR‐17‐5p^34^ and human amniotic mesenchymal stem cells miR‐320a^35^ can restore ovarian vitality, increase the number of follicles, restore hormone levels, and improve POI. Therefore, the identification of exosome‐mediated cell–cell interaction may provide a new strategy for the clinical treatment of POI. Therefore, we determined to identify the difference of miRNAs in exosomes derived from ovarian tissues of POI mice and normal mice.

The high‐throughput sequencing of ovarian exosomes in the POI group and control group showed that there were significant differences in the expression of various miRNAs, miR‐141‐3p, and miR‐122‐5p in ovarian exosomes in chemotherapy group were significantly upregulated, among which the apoptosis‐related miR‐122‐5p was upregulated more than 32 times in ovarian exosome in the chemotherapy group. Previous studies have shown that the enhanced expression of miR‐141‐3p suppressed the proliferation and migration of ectopic ESCs and promoted their apoptosis via targeting KLF‐12.^36^ In addition, the study showed that miR‐141‐3p was dramatically decreased in the ovaries of rat PCOS models. Researchers transfected miR‐141‐3p mimics, and the results showed that miR‐141‐3p inhibited the apoptosis of rat ovarian GCs.^37^ However, our research contradicts it, so we choose to study miR‐122‐5p. As a specific miRNA molecule of liver cells, it accounts for nearly 70% of the total miRNA. It is known that miR‐122‐5p can inhibit tumors, and its expression in hepatocellular carcinoma, breast carcinoma, endometrial carcinoma, and ovarian cancer are significantly reduced, so overexpression of miR‐122‐5p can reduce the tumorigenicity of cancer cells.^38^ In addition, miR‐122‐5p plays an important role in maintaining liver development and regulating cholesterol biosynthesis,^39^ and it also plays a vital role in stabilizing HBV and HCV mRNA and virus transmission.^29^

Besides, we selected the target factors BCL9 and TAP‐63, and the results of qPCR showed that miR‐122‐5p could downregulate the target factor BCL9, while TAP‐63 was the opposite, so we chose to study BCL9. BCL9 is highly expressed in human tumor tissues, and β‐catenin/BCL9 is an important target for cancer treatment. However, the relationship between miR‐122‐5p target gene BCL9 and its protein expression and POI has not been reported in the literature. Our research shows that miR‐122‐5p in ovarian tissue‐derived exosomes promotes GC apoptosis by regulating the expression of BCL9, and it is speculated that POI can be improved by regulating ovarian GCs apoptosis. In the future study, the effects of miR‐122‐5p on ovarian function will be detected in vivo.

In this study, our results suggest that ovarian tissue exosomal miRNAs is involved in the regulation of POI after chemotherapy. Clarifying the function and mechanism of miR‐122‐5p in chemotherapy‐induced ovarian damage will help to reveal the molecular regulation mechanism of POI, and provide a new target for protecting ovarian function. Exosomes play a key role in restoring ovarian function induced by CTX, which mainly regulates the expression of BCL9 by delivering miR‐122‐5p to ovarian GCs, thus intervening with apoptosis. Our current study clarified the potential molecular mechanism of ovarian function recovery and provided new strategies and directions for POI treatment.

## CONFLICT OF INTEREST

The authors declare no conflict of interest.

## AUTHOR CONTRIBUTIONS

Xiujuan Zhang, Ruihong Zhang, and Yu‐Lan Mu conceived and designed the experiments; Xiujuan Zhang, Ruihong Zhang, Jing Hao, performed the experiments; Xiaoyan Huang, Ming Liu, and Mengxiao Lv analyzed the data; Xiujuan Zhang, Ruihong Zhang wrote the paper. Jing Hao and Yu‐Lan Mu reviewed and edited the paper.

## ETHICS STATEMENT

The protocol was reviewed and approved by the Medical Ethics Committee of the Shandong Provincial Hospital Affiliated to Shandong First Medical University.

## Data Availability

The data that support the findings of this study are available from the corresponding author upon reasonable request.
